# Supplementary material for: New immunomodulatory treatment protocol for canine leishmaniosis reduces parasitemia and proteinuria
Source: PLoS Negl Trop Dis. 2024 Dec 19;18(12):e0012712. doi: 10.1371/journal.pntd.0012712 (PMC11698568; doi:10.1371/journal.pntd.0012712)
Supplement: S1 Trial — (PDF) [file pntd.0012712.s002.pdf]

BIOIBERICA

GALILEI

(DoG triAL with Impromune in LEIshmania)

Clinical trial on the comparative efficacy and safety of adding IMPROMUNE® to the standard treatment protocol for canine leishmaniosis

STUDY NUMBER:	PJ-00002-18-BIO
Projected Start Date:	FEBRUARY 2019
Projected Finish Date:	DECEMBER 2022
Sponsor:	BIOIBÉRICA, S.A.U.

Signatory List

By signing this page the signatories agree to conduct the study according to the study protocol, appropriate standard operating procedures (SOPs) and legal requirements.

APPROVAL

MIRO CORRALES MARIA GUADALUPE - DNI 05378854M

Firmado digitalmente por MIRO CORRALES MARIA GUADALUPE - DNI 05378854M
Nombre de reconocimiento (DN): c=ES, o=UNIVERSIDAD COMPLUTENSE DE MADRID, ou=CERTIFICADO ELECTRONICO DE EMPLEADO PUBLICO, ou=05378854M, serialNumber=IDCES-05378854M, sn=MIRO CORRALES, givenName=MARIA GUADALUPE, cn=MIRO CORRALES MARIA GUADALUPE - DNI 05378854M
Fecha: 2018.12.05 17:06:39 +01'00'

DATED ____________ SIGNED ____________

Guadalupe Miró PhD

Lead Investigator

DATED 04/DEC/2018 SIGNED ____________

Laia Montell PhD

Study Manager

DATED 5/12/18 SIGNED ____________

Sergi Segarra PhD

Project Leader

DATED 10/12/18 SIGNED ____________

Daniel Martínez PhD

Head of Late Development and Product Support

DATED 04/DEC/2018 SIGNED ____________

Joan Gispert MD

R&D Director

Contents

1 Responsibilities Page 4

2 Independent advisory team Page 4

3 Study Title Page 5

4 Study Objective and Justification Page 5

5 Hypothesis Page 6

6 Test Substance and Placebo Page 7

7 Animals Page 11

8 Management Page 11

9 Site Description/Specification Page 11

10 Study Design Page 12

11 Objectives Page 13

11.1 Primary objective Page 13

11.2 Secondary objective Page 13

12 Procedures Page 14

12.1 Inclusion/exclusion criteria Page 14

12.1.1 Inclusion criteria Page 14

12.1.2 Exclusion criteria Page 14

12.2 Criteria of evaluation Page 15

13 Study Visits Page 17

14 Sample collection and processing Page 21

14.1 Sample collection Page 21

14.2 Sample processing Page 21

14.3 Sample shipment Page 21

15 Safety Page 21

16 Statistics Page 22

17 Publication Page 23

18 References Page 24

1 Responsibilities

Lead Investigator	Guadalupe Miró Corrales DVM, PhD, Dipl. EVPC<br>Catedrática del Departamento de Sanidad Animal<br>Responsable Consulta Patología Infecciosa y Parasitaria del Hospital Clínico Veterinario<br>Facultad de Veterinaria<br>Universidad Complutense de Madrid<br>gmiro@ucm.es
Laboratory Leader	José Joaquín Cerón, DVM, PhD, Dipl ECVCP<br>Universidad de Murcia
Study Manager	Laia Montell PhD<br>R&D Study Manager<br>Late Development & Product Support<br>BIOIBÉRICA, S.A.U.<br>Barcelona<br>lmontell@bioiberica.com
Project Leader	Sergi Segarra PhD<br>R&D Project Leader<br>Late Development & Product Support<br>BIOIBÉRICA, S.A.U.<br>Barcelona<br>ssegarra@bioiberica.com

2 Independent advisory team

- Lluís Ferrer, BVSc, PhD, Dipl ECVD

  Universitat Autònoma de Barcelona (UAB)

- Xavier Roura, DVM, PhD, Dipl ECVIM-CA

  Hospital Clínic Veterinari

  Universitat Autònoma de Barcelona (UAB)

- Laia Solano, Professora agregada, DVM, PhD, Dip. ECVCP

  Departament de Medicina i Cirurgia Animals, Facultat de Veterinària (UAB)

  Servei Patologia Clínica, Hospital Clínic Veterinari (UAB)

3 Study Title

Clinical trial on the comparative efficacy and safety of adding IMPROMUNE to the standard treatment protocol for canine leishmaniosis

4 Study Objective and Justification

- **Canine leishmaniosis (CanL)** caused by *Leishmania infantum* is a major global zoonosis potentially fatal to dogs and humans. Prevalence of infection in dogs can be as high as 50–80% in Mediterranean countries, while prevalence of disease varies from 2 to 5%. Currently, the most effective treatment against CanL consists of subcutaneous N-methylglucamine antimoniate (MGA) for 4-6 weeks combined with oral allopurinol for at least 6 months. This combination of drugs with leishmanicidal (MGA) and leishmaniostatic (allopurinol) effects is considered as the standard treatment and currently recommended by the LeishVet guidelines.[1] It has been shown to reduce parasite load and to allow a state of remission from clinical disease over prolonged periods of time. However, nowadays neither this treatment nor any other have achieved a 100% cure in dogs. Besides that, these drugs have side effects:
  - **Allopurinol** can cause xanthinuria, which can develop as early as 3 weeks after starting treatment, and can occur in a high number of cases (31% reported by Torres et al. 2016;[2] and 41% by Segarra et al. 2017)[3]. Xanthinuria can lead to urolithiasis and renal mineralization.
  - **MGA** is potentially nephrotoxic[4]. In dogs undergoing MGA treatment, with or without allopurinol, development of signs of renal disease, such as increased creatinine and proteinuria have been reported.[5,6] Moreover, these negative effects on kidney function can further exacerbate the signs of renal disease caused by CanL, as glomerulonephritis is a common finding in patients suffering from this disease.[7]
  - **Impromune** (Bioiberica, SAU), a combination of nucleotides and Active Hexose Correlated Compound (AHCC), modulates the immune response and has been proven to be efficacious in CanL patients in two clinical studies (PRJ-00122):

    - STUDY 1: Multicentre, open-label, positively-controlled randomized clinical trial in dogs with clinical leishmaniosis receiving an initial 28-day course of MGA, comparing the effects of Impromune *vs* allopurinol administration for 6 months. The administration of Impromune achieved a better clinical efficacy than allopurinol (significantly lower clinical score after 6 months) without

promoting xanthinuria (0% of cases, compared to 41% of cases in the allopurinol group). Parasite load (RT-PCR) was reduced with both treatments and cellular immunity was also improved in both groups.[3]

- STUDY 2: Multicentre, double-blinded, placebo-controlled randomized clinical trial in clinically healthy *Leishmania*-infected dogs. After 365 days, a significantly lower proportion of dogs showed disease progression in the Impromune group (3/20; 15%), compared to the placebo group (10/22; 45.5%). ELISA serology was reduced only with Impromune and the mean clinical score of disease severity was significantly lower in the supplement group after 180 days. It was also a useful study to confirm the safety of non-stop one-year administration of Impromune.[8]

Besides that, so far, Impromune has been shown to be a safe product and no side effects on renal or urinary function have been reported.

- **Adding Impromune to the standard treatment protocol** could enhance the effective immune response in CanL patients, and hence increase its efficacy. It could also reduce its side effects by reducing the need for MGA cycles. Given that MGA treatment requires daily injections, this would also result in an easier treatment protocol for owners as well as an improved owner compliance.

5 Hypothesis

The **new treatment protocol** presented herein, consisting of a **combination of MGA with allopurinol and Impromune**, is more efficacious than the currently recommended standard treatment, which combines MGA and allopurinol.

More specifically, we expect Impromune to allow a significant reduction in the disease relapses over 2 years in each study group.

6 Test Substance and Placebo

Test Substance (IMPROMUNE)

Product:	IMPROMUNE (F1 592)
Manufacturer's name:	Bioibérica S.A.U., Barcelona, Spain
Composition:	585 mg Nucleoforce Dogs (Bioiberica SAU, Barcelona, Spain)<br>315 mg AHCC (Amino Up Chemical Co. Ltd., Sapporo, Japan)<br>678 mg microcrystalline cellulose<br>144 mg flavourings<br>15 mg colloidal anhydrous silica<br>13 mg magnesium stearate
Handling & Storage:	Store at room temperature away from moisture and heat.
Batch Number:	TBD
Shelf Life:	2 years
Dosage form:	Tablet
Dose tested:	Once daily following the recommended weight-based dosing:<br><10 kg: 1/2 tablet<br>11-25 kg: 1 tablet<br>≥26 kg: 2 tablets
Mode of administration:	Oral administration
Indication:	Modulation of the immune response in companion animals

Placebo

Product:	PLACEBO
Manufacturer's name:	Bioibérica S.A.U.
Composition:	1428 mg microcrystalline cellulose<br>144 mg flavourings<br>15 mg colloidal anhydrous silica<br>13 mg magnesium stearate
Handling & Storage:	Store at room temperature away from moisture and heat.
Batch Number:	TBD
Shelf Life:	2 years
Dosage form:	Tablet
Dose tested:<br>weight-based dosing:	Once daily following the same Impromune recommended<br><br><10 kg: 1/2 tablet<br>11-25 kg: 1 tablet<br>≥26 kg: 2 tablets
Mode of administration:	Oral administration
Indication:	Study placebo

Glucantime (standard treatment)

Product:	GLUCANTIME
Manufacturer's name:	Merial Laboratorios S.A., Barcelona, Spain
Composition:	MGA 300 mg/ml
Handling & Storage:	According to manufacturer's intructions
Batch Number:	TBD
Shelf Life:	According to manufacturer's intructions
Dosage form:	Injectable solution
Dose tested:	50 mg/kg every 12h for 28 days.
Mode of administration:	Subcutaneous injection in different locations
Indication:	Treatment of CanL (leishmanicidal)

Allopurinol (standard treatment)

Product:	ZYLORIC
Manufacturer's name:	FAES FARMA, S.A. Vizcaya, Spain
Composition:	Allopurinol 100/300 mg
Handling & Storage:	According to manufacturer's intructions
Batch Number:	TBD
Shelf Life:	According to manufacturer's intructions
Dosage form:	Tablets
Dose tested:	10 mg/kg every 12h
Mode of administration:	Oral administration
Indication:	Treatment of CanL (leishmaniostatic)

7 Animals

Species and breed	Dogs of various breeds with confirmed naturally-occurring leishmaniosis.<br><br>Diagnosis of CanL will be confirmed by compatible clinical signs and laboratory analyses: positive ELISA serology plus positive PCR result from whole blood.
Age	Different ages
Sex	Male and females not intended for breeding during the study period
Origin	Client owned veterinary patients
Identification	Dogs participating in the study will be privately owned. Dogs will be identified by their given name. In addition, each animal will be assigned a unique case number, assigned on enrolment for study identification purposes.

8 Management

Feed	The dogs will be fed a regular diet which ensures a correct nutritional intake. Different trademarks and formulations could be used but they will be excluded if they were receiving any kind of specific diet or supplements to improve their immune response (Canine Leishmaniasis Management Affinity).
Water	Owners normal practice
Housing	Dogs will be in their normal domestic environment and arrangements. Dogs may be housed with or without other animals.

9 Site Description/Specification

The study will be conducted at the Veterinary clinics/hospitals located in countries where CanL is endemic, including Spain and Portugal.

10 Study Design

This will be a multicenter, randomized, double-blind, placebo-controlled clinical trial.

MGA administration (50 mg/kg every 12h) will be performed initially (day 0 to day 28) and, after that, MGA cycles will be administered only when deemed necessary in accordance with the veterinary and principal investigator decision.

Ethics approval of the study protocol will be managed through a qualified and competent Committee of Research Ethics.

Animals will be assigned to one of two treatment groups: control group (standard treatment plus placebo) or active group (standard treatment plus Impromune).

Treatment	Total Animals per Treatment
MGA day 0 to 28 + Allopurinol day 0 to 365 + PLACEBO day 0 to day 730.	40 dogs
MGA day 0 to 28 + Allopurinol day 0 to 365 + IMPROMUNE day 0 to day 730.	40 dogs

Both the active product and the placebo will be presented in boxes containing 20 tablets each.

Labelling
The labelling of the tested product will include information in accordance to the regulatory requirements applicable.

Randomization and blinding
One hundred and thirty-six dogs will be enrolled onto the study and will be randomly assigned to one of two groups: active or placebo, after verifying the fulfillment of the inclusion and exclusion criteria.

Each treatment will be assigned a code (A or B) in order to restrict the knowledge of the treatment group assignments. The treatment codes knowledge will be restricted and kept by an investigator not involved

in the project until the end of the study, and both the dogs' owners and evaluators (including clinicians and those analyzing samples and tissues) will have no knowledge of the treatment group assignments.

The study nursing staff will receive the treatment assignation and will act as dispenser.

The appearance of both treatments will be identical and will be presented in identical containers. Randomization will be carried out with the software SPSS for windows (IBM).

11 Objectives

11.1 Primary objective

To evaluate the efficacy of a new treatment protocol for CanL consisting of a combination of MGA, allopurinol (the first year) and Impromune over 2 years in sick dogs with naturally-occurring *Leishmania* disease, compared to the standard treatment.

11.2 Secondary objective

- To assess the potential MGA-sparing effect of adding Impromune to the current standard protocol for treating CanL.
- To evaluate the beneficial effects of this new treatment protocol, compared to those of the current one, in terms of clinical signs and clinicopathological abnormalities, parasite load and antibody levels.
- To investigate the changes in the immune response exerted by the addition of Impromune to the current standard treatment for CanL.
- To evaluate the safety.

12 Procedures

12.1 Inclusion/exclusion criteria

12.1.1 Inclusion criteria

1. Clinical signs and/or clinicopathological abnormalities associated with CanL. Dogs will be classified in stage II or III based on clinical signs and/or clinicopathological abnormalities and serological status according to the LeishVet Clinical Staging of CanL (Solano-Gallego et al. 2009)[1], plus:

2.a. Seropositivity by *Leishmania* confirmation by positive TRFIA (Time-Resolved Fluorescence Immunoassay) result (high antibody levels, defined as a 3-4 fold elevation above the cut off level of a well established reference laboratory)

OR

2.b. Seropositivity by *Leishmania* confirmation by positive TRFIA (Time-Resolved Fluorescence Immunoassay) result (medium titration) plus positive PCR from blood sample.

12.1.2 Exclusion criteria

Dogs with the following criteria or concurrent diseases will be excluded:

- Vaccination against CanL, regardless of the type and moment of vaccination.
- Treatment with allopurinol, MGA, miltefosine, domperidone, cyclosporine or glucocorticoids or other immunomodulating or immunosuppressive drug (Cytopoint, oclacitinib, azathioprine, mofetil, mycophenolate, etc.) 4 months before entering the study.
- Dogs receiving any kind of special diet or supplements to improve their immune response.
- Dogs affected with CKD (IRIS ≥ 3)
- Pregnant and lactating bitches.

12.2 Criteria of evaluation

Main efficacy variable:

Percentage of the number of the disease relapses over 2 years in each study group.

In a situation where the subjects show clinical signs and/or clinicopathological abnormalities compatible with canine leishmaniosis, the whole analysis must be performed in order to elucidate if it is a disease relapse (ELISA and PCR):

1. Clinical signs and/or clinicopathological abnormalities associated with CanL. Dogs will be classified in stage II or III based on clinical signs and/or clinicopathological abnormalities and serological status according to the LeishVet Clinical Staging of CanL (Solano-Gallego et al. 2009)[1], plus:

2.a. Seropositivity by Leishmania confirmation by positive TRFIA (Time-Resolved Fluorescence Immunoassay) result (high antibody levels, defined as a 3-4 fold elevation above the cut off level of a well established reference laboratory)

OR

2.b. Seropositivity by Leishmania confirmation by positive TRFIA (Time-Resolved Fluorescence Immunoassay) result (medium titration) plus positive PCR from blood sample.

Secondary efficacy variables:

- Clinical score including laboratory abnormalities (Miró et al. 2009)[9].
- LeishVet Clinical Staging of CanL (Solano-Gallego et al. 2009)[1].
- Antibody levels against *L. infantum*.
- Parasite load from blood sample (PCR)
- IRIS (International Renal Interest Society) staging of chronic kidney disease, based on creatinine levels and urine protein to creatinine ratio (UPC).
- Acute phase proteins (C-reactive protein, ferritin, paraoxonase-1 and haptoglobin).
- Survival rate.

Clinical signs	Score			
	0	1	2	3
Appetite	Normal	Slightly reduced (>half normal intake)	Markedly reduced (<half normal intake)	Anorexia
Apathy	Absent	Mild (active if stimulated)	Severe	Prostration
Polydipsia	Absent	Drinking less than 2x normal amount	Drinking between 2x and 4x normal amount	Drinking more than 4x normal amount
Temporal muscle atrophy	Absent	Mild	Severe	Temporal muscles not visible
General muscle atrophy	Absent	Mild (emaciation)	Severe	Cachexia
Lymphadenomegaly	-	1 or 2 enlarged lymph nodes	> 2 enlarged lymph nodes	Generalized lymphadenomegaly
Splenomegaly	-	-	Splenomegaly	-
Conjunctivitis and/or blepharitis	Absent	Mild unilateral	Bilateral / severe unilateral	Bilateral severe
Keratitis / uveitis	Absent	Mild unilateral	Bilateral / severe unilateral	Bilateral severe
Pale mucous membranes	Absent	Mild	Moderate	Severe
Oral mucosa	Normal	1 or 2 small ulcers and/or nodules	> 2 small ulcers and/or nodules	> ¼ surface ulcerated or with nodules
Nasal mucosa	Normal	-	Epistaxis	-
GI mucosa	Normal	Occasional vomiting/diarrhea	Frequent vomiting/diarrhea or occasional hematochezia	Frequent bloody vomiting/diarrhea or frequent hematochezia
Arthritis	Absent	1 joint affected	Polyarthritis in one limb	Polyarthritis in more than one limb
Skin erythema	Absent	<10% body surface or generalized but mild erythema	< 25% of body surface or generalized moderate erythema	> 25% of body surface
Skin ulcers	Absent	1 or 2 ulcers	3 to 5 ulcers	More than 5 ulcers
Skin nodules	Absent	1 or 2 nodules	3 to 5 nodules	More than 5 nodules
Alopecia +/- scaling/ exfoliation	Absent	< 10% of body surface or generalized but mild alopecia	< 25% of body surface or generalized moderate alopecia	> 25% of body surface
Onychogryphosis	Absent	Mild hypertrophy	Moderate hypertrophy	Severe hypertrophy

Clinical Scoring system. Adapted from Miró et al. 2009[9]

13 Study Visits

- **On-site visits:** Study subjects will attend the recruiting center at different time points during the study (days 0, 30, 180, 365, 545 and 730) for a complete physical examination, scoring of clinical signs, clinical staging of CanL and collection of blood and urine.

- **Phone interviews:** Between the on-site visits, investigators will contact the owners by phone at different time points: days 90, 270, 455 and 635. This interview will allow the clinician to ask the owner how everything is going, and to decide whether an extra on-site visit is needed in order to decide if an MGA cycle needs to be administered before the next visit.

  Therefore, if the owner suspects of any worsening in the clinical signs, an extra on-site visit will be performed.

- In addition, each owner will have a **Daily medication record**, where the owner will be able to highlight any observation and he/she will record the daily dose given.

ON-SITE VISITS

VISIT 1 (DAY 0)

- Demographic data: breed, age and sex.
- Daily medication record delivery.
- Next visit schedule.
- Complete physical examination (measurement of body temperature, blood pressure and weight)
- Scoring of clinical signs: Clinical Score (Miró et al. 2009)[9]
- LeishVet Clinical Staging of CanL (Solano-Gallego et al. 2009)[1]
- Collection of blood:
  - **Whole blood:** complete blood count (CBC), PCR
  - **Serum:** serum biochemistry, serum protein electrophoresis, antibody levels against *L. infantum*, acute phase proteins (C-reactive protein, ferritin, paraoxonase-1 and haptoglobin).
- Collection of urine: organoleptic features, whole urine analysis, sediment analysis, protein and creatinine (IRIS staging of chronic kidney disease).

VISIT 2 (DAY 30)

- Daily medication record review.
- Next visit schedule.
- Complete physical examination (measurement of body temperature, blood pressure and weight)
- Scoring of clinical signs: Clinical Score (Miró et al. 2009)[9]
- Collection of blood:
  - **Whole blood:** complete blood count (CBC)
  - **Serum:** serum biochemistry, serum protein electrophoresis, antibody levels against *L. infantum*, acute phase proteins (C-reactive protein, ferritin, paraoxonase-1 and haptoglobin).
- Collection of urine: organoleptic features, whole urine analysis, sediment analysis, protein and creatinine (IRIS staging of chronic kidney disease).

VISIT 4 (DAY 180)

- Daily medication record review.
- Next visit schedule.
- Complete physical examination (measurement of body temperature, blood pressure and weight)
- Scoring of clinical signs: Clinical Score (Miró et al. 2009)[9]
- Collection of blood:
  - **Whole blood:** complete blood count (CBC), PCR
  - **Serum:** serum biochemistry, serum protein electrophoresis, antibody levels against *L. infantum*, acute phase proteins (C-reactive protein, ferritin, paraoxonase-1 and haptoglobin).
- Collection of urine: organoleptic features, whole urine analysis, sediment analysis, protein and creatinine (IRIS staging of chronic kidney disease).

VISIT 6 (DAY 365)

- Daily medication record review.
- Next visit schedule.
- Complete physical examination (measurement of body temperature, blood pressure and weight)
- Scoring of clinical signs: Clinical Score (Miró et al. 2009)[9]
- Collection of blood:
  - **Whole blood:** complete blood count (CBC), PCR

  - **Serum:** serum biochemistry, serum protein electrophoresis, antibody levels against *L. infantum*, acute phase proteins (C-reactive protein, ferritin, paraoxonase-1 and haptoglobin).
- Collection of urine: organoleptic features, whole urine analysis, sediment analysis, protein and creatinine (IRIS staging of chronic kidney disease).

VISIT 8 (DAY 545)

- Daily medication record review.
- Next visit schedule.
- Complete physical examination (measurement of body temperature, blood pressure and weight)
- Scoring of clinical signs: Clinical Score (Miró et al. 2009)[9]
- Collection of blood:
  - **Whole blood:** complete blood count (CBC)
  - **Serum:** serum biochemistry, serum protein electrophoresis, antibody levels against *L. infantum*, acute phase proteins (C-reactive protein, ferritin, paraoxonase-1 and haptoglobin).
- Collection of urine: organoleptic features, whole urine analysis, sediment analysis, protein and creatinine (IRIS staging of chronic kidney disease).

VISIT 10 (DAY 730)

- Daily medication record review.
- Complete physical examination (measurement of body temperature, blood pressure and weight)
- Scoring of clinical signs: Clinical Score (Miró et al. 2009)[9]
- LeishVet Clinical Staging of CanL (Solano-Gallego et al. 2009)[1]
- Collection of blood:
  - **Whole blood:** complete blood count (CBC), PCR
  - **Serum:** serum biochemistry, serum protein electrophoresis, antibody levels against *L. infantum*, acute phase proteins (C-reactive protein, ferritin, paraoxonase-1 and haptoglobin).
- Collection of urine: organoleptic features, whole urine analysis, sediment analysis, protein and creatinine (IRIS staging of chronic kidney disease).

VISITS CHRONOGRAM

	**V1**<br>Day 0	**V2**<br>Day 30<br>(1 m)	**V3**<br>Day 90<br>(3 m)	**V4**<br>Day 180<br>(6 m)	**V5**<br>Day 270<br>(9 m)	**V6**<br>Day 365<br>(12 m)	**V7**<br>Day 455<br>(15 m)	**V8**<br>Day 545<br>(18 m)	**V9**<br>Day 635<br>(21 m)	**V10**<br>Day 730<br>(24 m)
	**Start Tx**		**Phone Interview**		**Phone Interview**		**Phone Interview**		**Phone Interview**	**Finish Tx**
Physical examination	x	x		x		x		x		x
Clinical score	x	x		x		x		x		x
LeishVet Clinical Staging of CanL	x									x
-PROP: Informed Consent<br>-VET: NDA	x									
Daily medication record delivery / review	x	x		x		x		x		x
Next visit schedule	x	x		x		x		x		x
CBC	x	x		x		x		x		x
Parasite load evaluation (PCR)	x			x		x				x
Biochemistry and protein electrophoresis	x	x		x		x		x		x
Antibody levels against *L. infantum*	x	x		x		x		x		x
Acute phase proteins	x	x		x		x		x		x
Urinalysis	x	x		x		x		x		x

14 Sample collection and processing

14.1 Sample collection

Blood (3 mL EDTA whole blood (one tube with 1 ml and another tube with 2ml) + 2 mL serum) and urine (4 mL) samples will be collected at baseline, and after 30, 180, 365, 545 and 730 days of treatment, and sent to the appropriate lab.

14.2 Sample processing

- Blood and urine samples will be analyzed at the Interlab-UMU, University of Murcia:
  - **Parameters from whole blood**: complete blood count (CBC), real time-PCR (RT-PCR) parasite load evaluation.
  - **Parameters from serum**: serum biochemistry, serum protein electrophoresis, and antibody levels against *L. infantum* (TRFIA: Time-Resolved Fluorescence Immunoassay), acute phase proteins (C-reactive protein, ferritin, paraoxonase-1 and haptoglobin).
  - **Parameters from urine**: organoleptic features, whole urine analysis, sediment analysis, protein and creatinine.

14.3 Sample shipment

- The blood and urine samples will be sent by MRW to the following address:

  José Joaquín Cerón

  4ª planta Hospital Clínico Veterinario

  Facultad de Veterinaria

  Campus de Espinardo, 30100

  Universidad de Murcia

15 Safety

Any adverse event must be registered, related or not with the tested product.

- Alterations in hematology / biochemistry.
- Gastrointestinal disturbances or urinary abnormalities, with special attention to development of xanthinuria.
- Painful swelling at the site of MGA injection, anorexia, disturbances in locomotion and weariness.
- Cellulitis, nephrotoxicity, pancreatitis and/or fever.

16 Statistics

The statistic analysis will be performed by EURECAT, Centre Tecnològic de Catalunya.

- Baseline differences: Student's t-test for quantitative variables and Fisher's exact test for categorical variables.
- Treatment effects (differences between groups) will be assessed by analysis of covariance (ANCOVA) for quantitative variables and Fisher's exact test for categorical variables.
- For quantitative variables, changes over time within each group will be analyzed by repeated-measure analysis of variance (rmANOVA). For categorical variables, changes over time within each group will be analyzed with McNemar's test.
- Level of statistical significance will be set at 5%.

- There is insufficient information published that can be used for a proper calculation of the sample size based on the main efficacy variable, and it is difficult to make an estimate. Consequently, the sample size has been calculated based on previous publications, including the two clinical trials done with Impromune® which led to statistically significant effects. We have assumed a screening failure (SF) rate of 23% together with a drop-outs rate of 18%, according to the rates obtained in the previous clinical trial.

A start preliminary statistical analysis will be performed when 20 cases per group have finished the study in order to know if objective of the study will be achieved with the target sample size and the observed tendencies:

- If the preliminary analysis reveals that it is possible to achieve the objective of the study with the original target sample size, no additional actions are needed.
- If the preliminary analysis reveals that a sample size 15% larger than the original target is needed to achieve the objective of the study, it will be considered recruiting more cases.
- If the preliminary analysis reveals that a sample size of more than a 15% larger than the original target sample size is needed in order to achieve the objective of the study, it will be considered stopping the recruitment and evaluate the feasibility of the project.

17 Publication

At the end of the study, a Final Report will be performed which will include the whole data obtained from statistical analysis.

All data, information and results arising from the Study shall be the sole and exclusive property of Bioibérica, S.A.U.

Consequently, the investigators undertake to not use for its account or for the account of any third party, such data, information, or results.

Notwithstanding the foregoing, the investigators shall have the right to use, for its own research purposes, the results arising from the Study, subject to Bioibérica's prior written approval.

Biobérica will take the final decision about using the results of the study for publication and/or oral presentations in the scientific media and/or forums.

18 References

1. Solano-Gallego L, Koutinas A, Miro G, Cardoso L, Pennisi MG, Ferrer L, Bourdeau P, Oliva G, Baneth G. Directions for the diagnosis, clinical staging, treatment and prevention of canine leishmaniosis. *Vet Parasitol.* 2009, 165(1-2):1-18.

2. Torres M, Pastor J, Roura X, et al. Adverse urinary effects of allopurinol in dogs with leishmaniasis. *J Small Anim Pract.* 2016;57(6):299-304.

3. Segarra S, Miró G, Montoya A, et al. Randomized, allopurinol-controlled trial of the effects of dietary nucleotides and active hexose correlated compound in the treatment of canine leishmaniosis. *Vet Parasitol.* 2017;239:50-56.

4. Torres M, Bardagí M, Roura X, Zanna G, Ravera I, Ferrer L. Long term follow-up of dogs diagnosed with leishmaniosis (clinical stage II) and treated with meglumine antimoniate and allopurinol. *Vet J.* 2011;188:346-351.

5. Slappendel RJ, Teske E. The effect of intravenous or subcutaneous administration of meglumine antimonate (Glucantime®) in dogs with leishmaniasis. A randomized clinical trial. *Vet Q.* 1997;19(1):10-13.

6. Daza González M. Estudio de la enfermedad renal y la respuesta al tratamiento (antimoniales versus miltefosina) en perros con infección natural por leishmania infantum. Tesis Doctoral. 2016.

7. Baneth G, Shaw SE. Chemotherapy of canine leishmaniosis. *Vet Parasitol.* 2002;106(4):315-324.

8. Segarra S, Miró G, Montoya A, et al. Prevention of disease progression in Leishmania infantum -infected dogs with dietary nucleotides and active hexose correlated compound. *Parasites Vectors 2018 111.* 2018;11(1):103.

9. Miró G, Oliva G, Cruz I, et al. Multicentric, controlled clinical study to evaluate effectiveness and safety of miltefosine and allopurinol for canine leishmaniosis. *Vet Dermatol.* 2009;20(5-6):397-404.
