# Supplementary material for: New immunomodulatory treatment protocol for canine leishmaniosis reduces parasitemia and proteinuria
Source: PLoS Negl Trop Dis. 2024 Dec 19;18(12):e0012712. doi: 10.1371/journal.pntd.0012712 (PMC11698568; doi:10.1371/journal.pntd.0012712)
Supplement: Attachment — Submitted filename: renamed_7a7c4.docx [file pntd.0012712.s003.docx]

Animal Health Department

Veterinary Faculty,

Universidad Complutense de Madrid,
Avda. Puerta de Hierro, s/n. 28040 Madrid, Spain.

Camila I. de Oliveira
Academic Editor PLOS Neglected Tropical Diseases

Hira Nakhasi
Section Editor PLOS Neglected Tropical Diseases

9^th^ June 2024

Dear editors,

Thank you for your email dated 24^th^ April 2024. We really appreciate the constructive and helpful feedback from the editors and the reviewers.

We are sending you the revised manuscript PNTD-D-24-00363 “*New immunomodulatory treatment protocol for canine leishmaniosis reduces parasitemia and proteinuria*” for publication in *PLOS Neglected Tropical Diseases*.

Revisions in the manuscript are shown using track changes.

Please find below the responses to the comments (in italics),

**Reviewer's Responses to Questions**

**Reviewer #1:**

- Are the objectives of the study clearly articulated with a clear testable hypothesis stated? Yes.
- Is the study design appropriate to address the stated objectives? No.
- Is the population clearly described and appropriate for the hypothesis being tested? No.
- Is the sample size sufficient to ensure adequate power to address the hypothesis being tested? Yes.
- Were correct statistical analysis used to support conclusions? Yes.
- Are there concerns about ethical or regulatory requirements being met? No.

*RESPONSE:*

*Thank you for reviewing our manuscript.*

*We see that you consider that the study design was not appropriate to address the stated objective and that the population was not clearly described and appropriate for the hypothesis being tested.*

*The objective of our study was to assess the efficacy and safety of a new immunomodulatory treatment protocol for CanL consisting of adding nucleotides and AHCC to the recommended standard MGA-allopurinol treatment. Therefore, we designed the study accordingly and recruited a population that we believed would serve for that purpose, described it in detail and used it to test our hypothesis. That is, as explained in the material and methods sections, a multicenter, randomized, double-blind, placebo-controlled clinical trial in dogs with confirmed diagnosis of leishmaniosis.*

*To improve our manuscript following your comments, and if you believe that these points are not yet appropriately addressed in our article, we would kindly request for you to please let us know what the limitations of study design are. Furthermore, it is not clear to us what further details are needed to properly describe dog population studied. The authors do not fully understand why the dog population is not appropriate for the hypothesis being tested. Therefore, more information regarding these questions will be important to improve the manuscript.*

**Reviewer #2:**

The study meticulously articulates its objectives and presents a well-defined, testable hypothesis, ensuring the study design is suitably tailored to achieve the stated goals. The chosen population aligns perfectly with the hypothesis, the sample size is aptly calculated to provide robust power, appropriate statistical methods bolster the conclusions, and all ethical and regulatory standards are scrupulously upheld.

*RESPONSE:*

*Thank you for reviewing our article. We appreciate your comments.*

**Reviewer #3:**

The authors were very careful regarding the methodology. There is only one issue regarding the analysis of parasite load using blood, but the authors duly justified it.

*RESPONSE:*

*Thank you for reviewing our article. We appreciate your comments.*

**Results**

**Reviewer #1:**

- Does the analysis presented match the analysis plan? No.
- Are the results clearly and completely presented? No.
- Are the figures (Tables, Images) of sufficient quality for clarity? No.

*RESPONSE:*

*Thank you for your comments.*

*We believe that the analyses presented matches the analysis plan but, of course, we are open to any suggestions that can help improving our paper.*

*Some figures have been adjusted (creatinine and total clinical score) and should now be clearer and easier to understand to the reader.*

*Based on your concerns, if you still do not agree, could you please consider suggesting how results and figures could be improved?*

**Reviewer #2:**

The analysis aligns seamlessly with the planned approach, and the results are articulated with commendable clarity and completeness. The visual aids, including tables and images, are of high quality, ensuring clear communication of data. However, a detailed discussion is warranted regarding the observed pattern in the IRIS stages within the supplement group, particularly concerning the potential indication of patient relapses over time.

*RESPONSE:*

*Based on your suggestion, we have completed the discussion regarding the evolution of the IRIS staging as follows lines 319-324.*

**Reviewer #3:**

Only in the creatinine graph could the authors draw lines indicating the minimum and maximum reference values, making the figure clearer for the reader.

*RESPONSE:*

*Following your comment, we have modified Figure 6 so that there are now lines indicating the minimum and maximum reference values for creatinine.*

**Discussion**

Line 273: the authors need to make it clear in this part of the text that the clinical stability of dogs for another year after interrupting the treatment protocol with MGA and Allopurinol occurs in this region of the European continent. This detail is important because in tropical regions the infection pressure is different due to climatic conditions, among other factors.

*RESPONSE:*

*Point taken. We have specified that the study was conducted in Spain and Portugal and the implications that this may have. See lines 276-279.*

**Conclusions**

**Reviewer #1:**

- Are the conclusions supported by the data presented? Yes.
- Are the limitations of analysis clearly described? Yes.
- Do the authors discuss how these data can be helpful to advance our understanding of the topic under study? No.
- Is public health relevance addressed? Yes.

*RESPONSE:*

*Following your comment, we added an extra sentence on the Conclusions paragraph to state that our data contribute to advance our understanding of the topic under study. See lines 370-372*

**Reviewer #2:**

The conclusions are well-grounded in the data provided, with a transparent acknowledgment of the analysis's limitations. The authors effectively discuss the data's contribution to advance our understanding of the subject, also addressing its public health significance. However, an in-depth exploration of the IRIS stage patterns in the supplement group would enrich the discussion, particularly in relation to the potential for patient relapses over time.

*RESPONSE:*

*Based on your suggestion, we have completed the discussion regarding the evolution of the IRIS staging.*

**Reviewer #3:**

The conclusions are in accordance with the results obtained and presented in the manuscript.

Editorial and Data Presentation Modifications?

**Reviewer #1:**

(No Response)

**Reviewer #2:**

- Line 72: The term "me+2thylglucamine antimoniate" should be corrected to "N-methylglucamine antimoniate."

*RESPONSE: Corrected. Thank you.*

- The acronym AHCC is not explained in the text; could you please provide its definition?

*RESPONSE:*

*In prior publications, AHCC appears described as “Active Hexose Correlated Compound”. However, from 2018, the manufacturers of AHCC (Amino Up Co., Ltd., Japan) decided to eliminate all spelled-out names of their brands which had previously been used for many years (e.g. Active Hexose Correlated Compound) for trademark protection purposes. From then on, and as requested by the manufacturer, AHCC is a brand and it should not be described other than by explaining the following: “AHCC is a standardized extract of cultured Lentinula edodes mycelia (ECLM) produced by Amino Up Co., Ltd. (Sapporo, Japan)”. This is how it appears n our manuscript, in line with recent scientific publications also mentioning AHCC.*

- Clarification is needed on whether sample analysis was centralized in one lab or conducted separately at the 13 veterinary centers.

*RESPONSE:*

*All samples were sent to the same lab and analyzed there. We added additional information in the manuscript: All laboratory analyses were performed at Interlab-UMU (University of Murcia, Murcia, Spain).*

- In Figure 4, the meaning of the units U.A. for the total clinical score is unclear. Please clarify this in the axis or figure caption.

*RESPONSE:*

*Following your recommendation, we modified Figure 4.*

**Reviewer #3:**

(No Response)

**Summary and General Comments**

**Reviewer #1:**

(No Response)

**Reviewer #2:**

The treatment combining MGA, Allopurinol, and supplements offers a promising alternative for managing canine leishmaniasis. The authors effectively detail how this regimen improves clinical and laboratory outcomes, emphasizing its oral administration convenience for pet owners.

**Reviewer #3:**

The authors recognize the limitations of the study, especially in relation to possible concomitant diseases in animals, which could have some impact on the results. However, any and all field studies in an endemic area are prone to factors of this nature. However, this does not reduce the relevance of the study, which was very well conducted.

We appreciate all your insightful comments.

Thank you for taking the time and energy to help improving the manuscript.

Looking forward to hearing from you soon.

Sincerely,

Prof. Guadalupe Miró.
