# Supplementary material for: New immunomodulatory treatment protocol for canine leishmaniosis reduces parasitemia and proteinuria
Source: PLoS Negl Trop Dis. 2024 Dec 19;18(12):e0012712. doi: 10.1371/journal.pntd.0012712 (PMC11698568; doi:10.1371/journal.pntd.0012712)
Supplement: Attachment — Submitted filename: renamed_64bc1.docx [file pntd.0012712.s005.docx]

**PNTD-D-24-00363R2 Miró et al., 2024**

**REBUTTAL LETTER TO REVIEWER 4**

Reviewer #4:

1- The objectives of the study clearly articulates with a testable hypothesis
2- The study design is appropriate to address the objectives
3- The population of the study is clearly described
4- The authors had a considerable lost in the number of animals, but in this kind of study is difficult to control this aspect.
5- Statistical analysis needs some clarification in relation to significance of results, since some parameters seem not to be significant and even in the characteristics of the population, there are some significant differences

--------------------

Reviewer's Responses to Questions: Reviewer #4:

Key Review Criteria Required for Acceptance?

As you describe the new analyses required for acceptance, please consider the following:

Methods

-Are the objectives of the study clearly articulated with a clear testable hypothesis stated?

-Is the study design appropriate to address the stated objectives?

-Is the population clearly described and appropriate for the hypothesis being tested?

-Is the sample size sufficient to ensure adequate power to address the hypothesis being tested?

-Were correct statistical analysis used to support conclusions?

-Are there concerns about ethical or regulatory requirements being met?

1- The objectives of the study clearly articulates with a testable hypothesis 2-The study design is appropriate to address the objectives

3- The population of the study is clearly described

4- The authors had a considerable lost in the number of animals, but in this kind of study is difficult to control this aspect.

5 Statistical analysis needs some clarification in relation to significance of results, since some parameters seem not to be significant and even in the characteristics of the population, there are some significant differences

RESPONSE: We reviewed the statistical analysis of the results and added an extra explanation in the Methods section (lines 174-175).

Results

1-Does the analysis presented match the analysis plan?

2-Are the results clearly and completely presented?

3-Are the figures (Tables, Images) of sufficient quality for clarity?

Reviewer #4:

1-Yes

2-Yes

3-needs to be more clear, some of them.

RESPONSE: We tried to make the tables and figures as clear as possible, so that the reader can follow and understand the results. During the review process, some of them have also been improved. However, we kindly ask you to let us know whether you still see how specifically we could improve them.

Conclusions

1-Are the conclusions supported by the data presented?

2-Are the limitations of analysis clearly described?

3-Do the authors discuss how these data can be helpful to advance our understanding of the topic under study?

4-Is public health relevance addressed?

1- Yes
2- Yes
3- Yes
4- Yes

Reviewer #4: The manuscript is interesting and shows the possibility to improve the treatment of Canine Visceral Leishmaniasis using supplements. Although it is not possible to define what is AHCC, it would be important to mention in since the abstract that is a supplement used in humans to improve the immune response. Another point, it would be discuss the possible mechanism why these compounds contribute to a better results in the outcome of the infection. Is it related to supression of the inflammatory responses? Increase the immune repsonses associated to a Th1 response? Etc

RESPONSE: Based on previous studies, we believe that the mechanism of action through which these compounds provide benefits in canine leishmaniosis is related to their ability to enhance the effective Th1 cellular immune response. This has been included in the Introduction (lines 98-100). However, following your comment, and in order to highlight this point, we added some extra information in the Discussion section (lines 328-329).

Lines 187-189 Neither were there differences (control vs supplement group, respectively) in LeishVet clinical stage (2.19±0.40 vs 2.37±0.49; p< 0.01) or total clinical score (9.00±5.58 vs 10.11±4.62; p< 0.01).

There are differences between the two groups concerning clinical stage and total clinical score

RESPONSE: Thank you for noticing. There was a mistake in those p-values. At baseline, there were indeed no differences between groups. We have corrected the p-values in the revised manuscript (lines 192-193).

Lines 225-226 A significant decrease over time (p= 0.004) in parasite load (blood qPCR) was seen only in the supplement group (Figure 5). It would be important to perform area under curve, because apparently, the difference is very low.

RESPONSE: There were actually no significant differences between groups. What we did find was that the decrease was only significant in the supplement group. As you will see, we re-wrote that statement in order t make it clearer (lines 228-231).

Lines 232- 233 We observe the same issue in the Figure 6 concerning the levels of creatinine, when the authors state a significant difference between the control and supplement groups that seems to occur in the first point before the end of treatment (30 days)

RESPONSE: Same as previous query - There were actually no significant differences between groups, so that is why AUC was not performed.

Summary and General Comments

The manuscript is interesting and shows the possibility to improve the treatment of Canine Visceral Leishmaniasis using supplements. Although it is not possible to define what is AHCC, it would be important to mention in since the abstract that is a supplement used in humans to improve the immune response. Another point, it would be discuss the possible mechanism why these compounds contribute to a better results in the outcome of the infection. Is it related to supression of the inflammatory responses? Increase the immune repsonses associated to a Th1 response? Etc

RESPONSE: Based on previous studies, we believe that the mechanism of action through which these compounds provide benefits in canine leishmaniosis is related to their ability to enhance the effective Th1 cellular immune response. This has been included in the Introduction (lines 98-100). However, following your comment, and in order to highlight this point, we added some extra information in the Discussion section (lines 328-329).

Lines 187-189 Neither were there differences (control vs supplement group,respectively) in LeishVet clinical stage (2.19±0.40 vs 2.37±0.49; p< 0.01) or total clinical score (9.00±5.58 vs 10.11±4.62; p< 0.01).

There are differences between the two groups concerning clinical stage and total clinical score

RESPONSE: Thank you for noticing. There was a mistake in those p-values. At baseline, there were indeed no differences between groups. We have corrected the p-values in the revised manuscript (lines 192-193).

Lines 225-226 A significant decrease over time (p= 0.004) in parasite load (blood qPCR) was seen only in the supplement group (Figure 5). It would be important to perform area under curve, because apparently, the difference is very low.

RESPONSE: There were actually no significant differences between groups. What we did find was that the decrease was only significant in the supplement group. As you will see, we re-wrote that statement in order to make it clearer (lines 228-231).

Lines 232- 233 We observe the same issue in the Figure 6 concerning the levels of creatinine, when the authors state a significant difference between the control and supplement groups that seems to occur in the first point before the end of treatment (30 days)

RESPONSE: Same as previous query - There were actually no significant differences between groups, so that is why AUC was not performed.
